# Late-onset lethal complication of non-surgically managed massive gastric conduit necrosis after esophagectomy: a case report

**DOI:** 10.1186/s40792-024-01955-1

**Published:** 2024-06-17

**Authors:** Hiroshi Takeuchi, Shuntaro Yoshimura, Mitsuhiro Daimon, Yasunobu Sakina, Yusuke Seki, Shintaro Ishikawa, Yoshiharu Kouno, Jo Tashiro, Seiji Kawasaki, Kazuhiko Mori

**Affiliations:** Department of Digestive Surgery, Mitsui Memorial Hospital, 1 Kanda Izumi-Cho, Chiyoda-Ku, Tokyo, 101-8643 Japan

**Keywords:** Gastric conduit necrosis, Esophagectomy, Case report

## Abstract

**Background:**

Gastric conduit necrosis (GCN) after esophagectomy is a serious complication that can prove fatal. Herein, we report a rare case of GCN with a severe course that improved with conservative treatment.

**Case presentation:**

We present the case of a 78-year-old male patient who underwent an Ivor Lewis esophagectomy and developed a massive GCN. The patient was critically ill in the initial phase but recovered quickly; he also had a ruptured gallbladder and a bleeding jejunal ulcer. On the 22nd postoperative day, massive GCN was revealed on endoscopy. Considering the recovery course, careful observation with a decompressing nasal gastric tube was the treatment of choice. The GCN was managed successfully, having been completely replaced by fine mucosa within 9 months postoperatively. The patient completed his follow-up visit 5 years after surgery without any evident disease recurrence. Five and a half years after the surgery, the patient presented with progressive weakness and deterioration of renal function. Gastrointestinal endoscopy revealed a large ulcer at the anastomotic site. Three months later, computed tomography revealed a markedly thin esophageal wall, accompanied by adjacent lung consolidation. An esophagopulmonary fistula was diagnosed; surgery was not considered, owing to the patient’s age and markedly deteriorating performance status. He died 2013 days after the diagnosis.

**Conclusions:**

Massive GCN after esophagectomy often requires emergency surgery to remove the necrotic conduit. However, this report suggests that a conservative approach can save lives and preserve the gastric conduit in these cases, thereby augmenting the quality of life.

## Background

Gastric conduit necrosis (GCN) after esophagectomy is a serious complication with fatal consequences if not detected and treated in time. Herein, we present the case of a 78-year-old male patient who underwent an Ivor Lewis esophagectomy and developed a massive GCN. In general, massive GCN after esophagectomy requires emergency surgery to remove the necrotic conduit. However, the case presented in this report suggests that a conservative approach may save lives as well as preserve the gastric conduit in such cases, thereby augmenting the quality of life.

## Case presentation

**Fig. 1:**
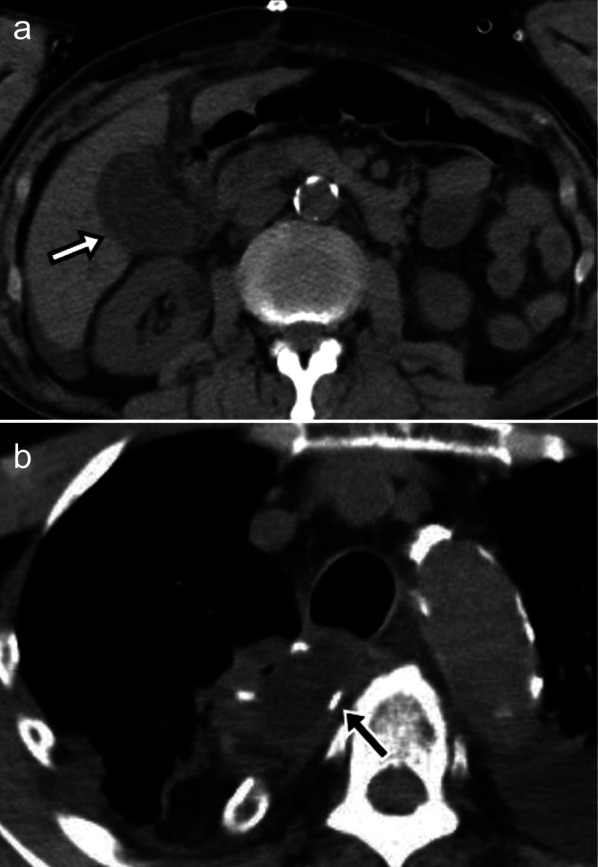
Computed tomography on the 4th postoperative day. **a** A medium-sized fluid collection is seen around the gallbladder (arrow). **b** No definite findings around the anastomotic site suggest anastomotic breakdown (arrow)

**Fig. 2:**
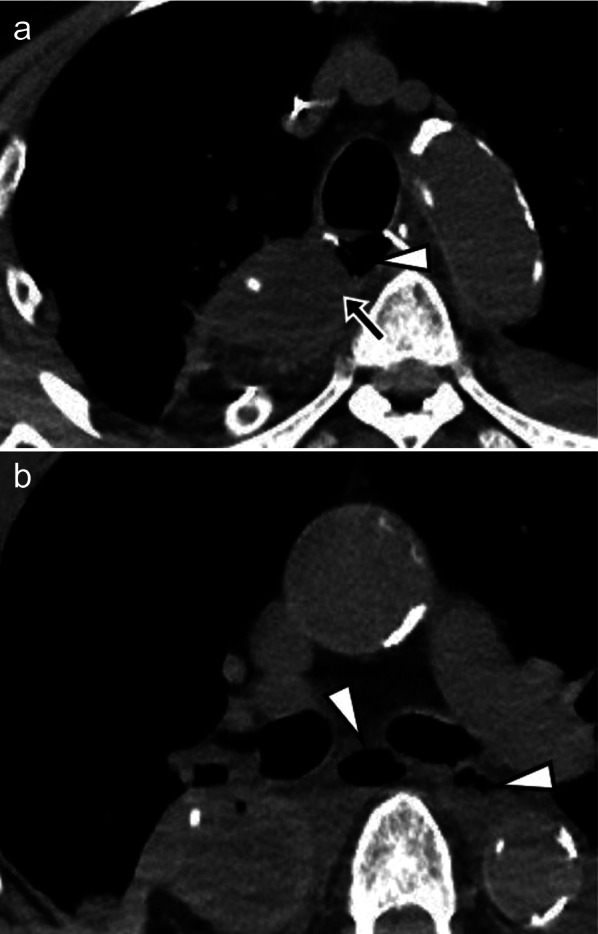
Computed tomography on the 6th postoperative day. **a**, **b** Free air (arrowhead) is seen around the anastomotic site (arrow)

A 78-year-old male patient presented with dysphagia caused by advanced esophageal squamous cell carcinoma (ESCC) of the lower thoracic esophagus. The disease was diagnosed as cT3N0, according to the Japanese Classification of Esophageal Cancer (11th Edition) [1]. The patient had no relevant medical history except for hypertension and moderate chronic kidney disease (Table 1). An Ivor Lewis esophagectomy was performed as a curative surgery. The postoperative course was uneventful before the acute onset of right hypochondrial pain on the 4th postoperative day (POD 4). Computed tomography (CT) revealed a moderate amount of fluid collection around the gallbladder (Fig. 1a); however, no remarkable abnormalities were observed around the anastomotic site (Fig. 1b). As he developed a high-grade fever on POD 6, his respiratory condition deteriorated steeply (PaO_2_: 68.8 mmHg at FiO_2_: 0.5). His mean blood pressure fell to 75 mmHg, and re-evaluation using CT revealed an anastomotic breakdown (Fig. 2). He was intubated and moved to the intensive care unit, where his general condition quickly improved after decompression of the gastric conduit using a nasogastric tube. While the patient was stable without notable pyrexia, thoracocentesis of the left chest revealed bile-containing pleural fluid on POD 8. The leaked bile also drained from the abdominal wound, and percutaneous transhepatic gallbladder drainage was performed on POD 9. The bile leakage subsided soon afterward. This episode was retrospectively diagnosed as a gallbladder rupture based on the histopathological findings of the resected gallbladder, which were obtained through open surgery performed on POD 128. The patient experienced another episode of gastrointestinal bleeding on POD 16 due to a jejunal ulcer forming near the site of the percutaneous feeding jejunostomy placed during the initial surgery. Bleeding was managed by an emergency operation to resect the bleeding area of the jejunum, revealing no other pathological conditions in the abdominal viscera, including the gallbladder.

**Table 1:** Preoperative laboratory and clinical findings

Laboratory data	
BUN	21 mg/dL
CREAT	1.66 mg/dL
AST	14 U/L
ALT	10 U/L
T-Bil	0.3 mg/dL
CEA	4.4 ng/mL
CA19-9	11.7 U/mL
SCC	4.4 ng/mL
Vital signs	
Blood pressure	116/50 mmHg
Heart rate	72 bpm/min
Echocardiography	Normal sinus rhythm, No axis deviation
Spirogram	
Forced vital capacity	3.1 L
Fraction of expiratory volume 1	0.59 L

*BUN* blood–urea–nitrogen, *T-bil* total bilirubin, *AST* aspartate aminotransferase, *ALT* alanine aminotransferase, *CEA* carcinoembryonic antigen, *CA19-9* carbohydrate antigen 19-9, *SCC* squamous cell carcinoma related antigen

**Fig. 3:**
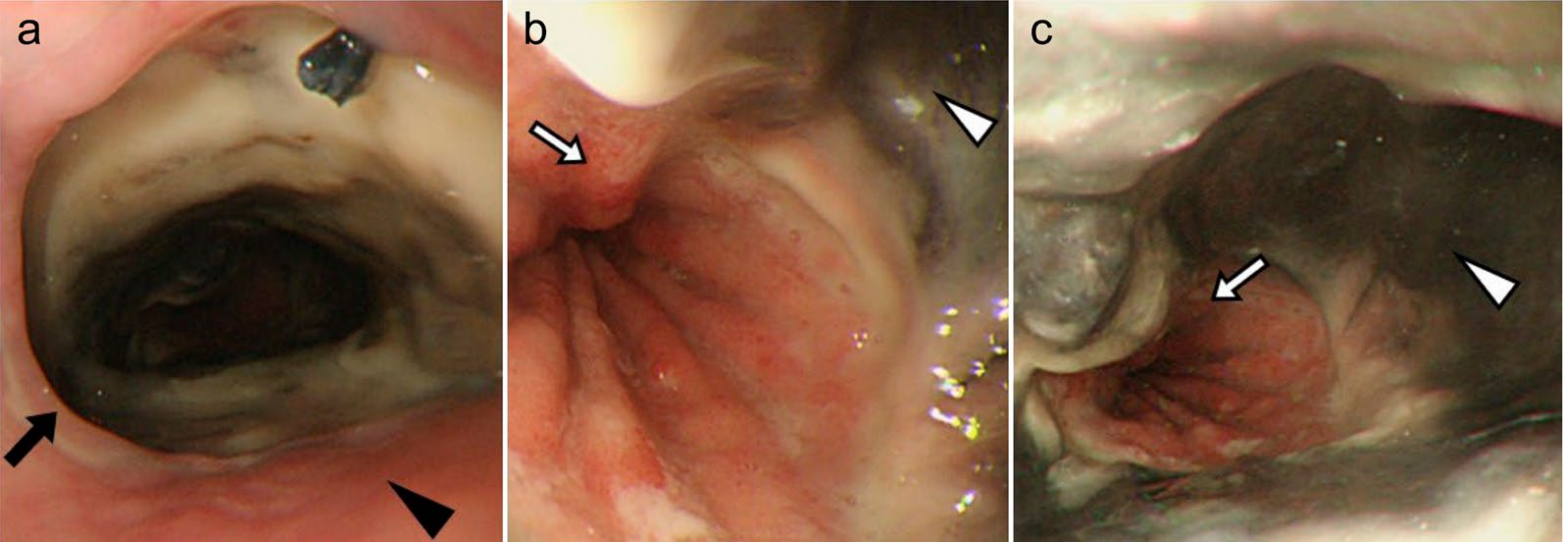
Upper gastrointestinal endoscopy on the 22nd postoperative day. Circular necrosis of the gastric conduit wall (white arrowhead), anastomotic site (black arrow), normal esophageal mucosa (black arrowhead), and gastric mucosa (white arrow) are seen

**Fig. 4:**
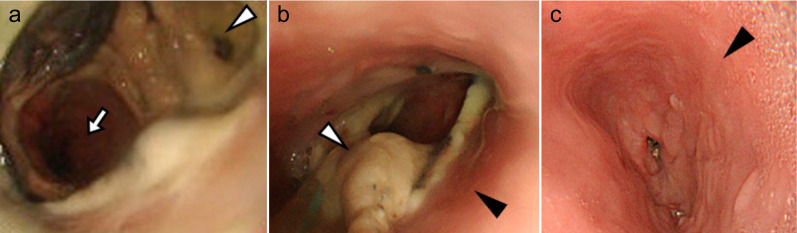
Upper gastrointestinal endoscopy. The necrotic black part of the conduit was gradually replaced by an ulcerous lesion and finally by regenerated mucosa on the 280th POD. **a** POD 36, **b** POD 90, **c** POD 280. White arrowhead: circular necrosis of the gastric conduit wall. Black arrowhead: normal esophageal mucosa. White arrow: gastric mucosa

The anastomotic leakage was considerably well-managed, and a contrast swallow was performed 16 days after the relook laparotomy, which revealed no contrast leakage. Before oral intake was restarted, an upper gastrointestinal endoscopy was performed on POD 22, which revealed circular necrosis of the gastric conduit wall extending 3 cm in length (Fig. 3). Careful observation with decompression via the nasogastric tube was regarded as a safer strategy than surgery, considering the patient’s recovery course. The intermittent suction pressure on the nasogastric tube was set at 50 cmH_2_O, and suction was continued until POD 36. The patient’s condition did not deteriorate, and enteral nutrition was initiated with a nasal nutritional tube placed proximal to the jejunum. Repeated evaluation by endoscopy on POD 36 showed partial replacement of the necrotic tissue by an ulcer, and the subsequent endoscopic findings revealed gradual improvements over 3 months (Fig. 4). Elective cholecystectomy and percutaneous feeding jejunostomy were performed on POD 128. He had been fed with 1400 kcal of polymeric formula by a nasal jejunal tube so far and by the percutaneous tube afterward. His serum nutritional marker recovered: albumin 3.2 g/dL on POD 164. Oral intake was permitted on POD 98, and repeated sessions of endoscopic dilatation for anastomotic strictures were required. After rehabilitation by a speech therapist, the patient was discharged on POD 183. The esophageal cancer was diagnosed as pT3N1 (11th Edition of Japanese Classification) [1]. Although several endoscopic interventions for balloon dilation were required, the anastomotic site was fully covered with regenerative mucosa (Fig. 4). The patient’s oral intake gradually improved, and the jejunostomy tube was removed 26 months postoperatively. His routine activities of daily living were well-restored, and he showed no evidence of cancer recurrence during 5 years of follow-up with regular CT surveillance.

**Fig. 5:**
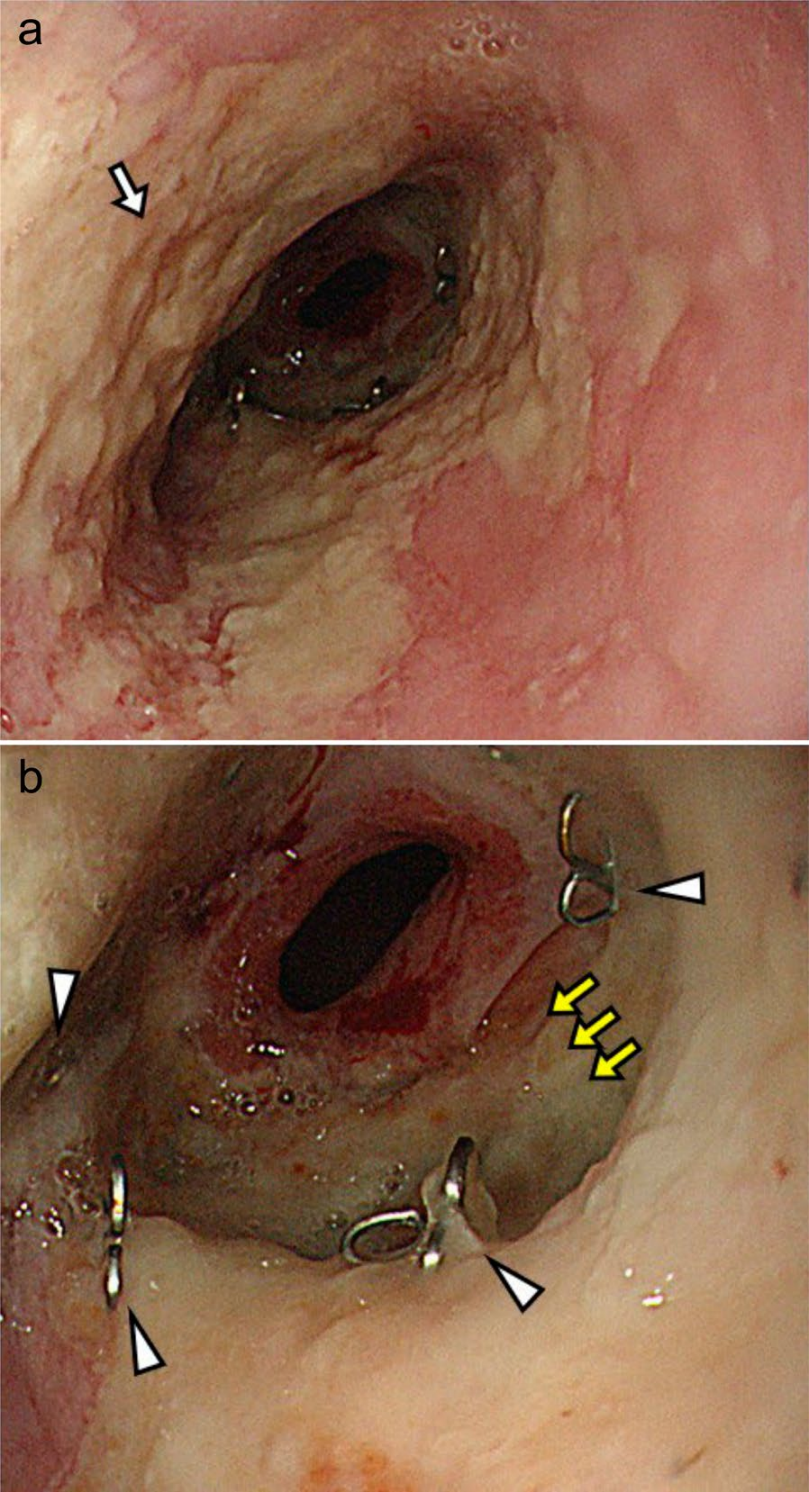
Upper gastrointestinal endoscopy 5 years and 4 months after surgery. A severe esophageal ulcer (white arrow) was observed with small metal staples (white arrowheads) indicating the anastomotic site. In the right-sided figure, the gap between the staples might have indicated a dehiscence of the anastomosis (yellow arrows)

**Fig. 6:**
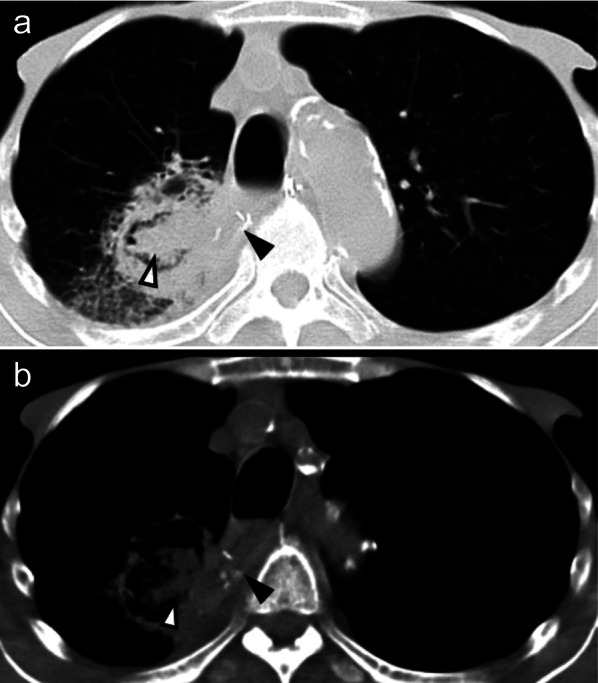
Computed tomography on the 5th postoperative year. **a**, **b** anastomotic sites (black arrowhead) and esophagopulmonary fistula (white arrowhead)

Meanwhile, the patient’s renal function slowly deteriorated, and a nephrologist followed him up. Hemodialysis was scheduled, and an arteriovenous shunt was created for vascular access. As his weakness worsened, an endoscopy was performed to evaluate the reasons for the patient’s worsening anorexia. A severe esophageal ulcer was observed, and reflux esophagitis was diagnosed; however, anastomotic dehiscence was retrospectively diagnosed (Fig. 5). CT revealed no anastomotic failure. Two months later, his weakness progressed with worsening loss of appetite. The patient was re-evaluated using CT, which revealed a markedly thin esophageal wall accompanied by an adjacent lung consolidation (Fig. 6). He was hospitalized with a diagnosis of an esophagopulmonary fistula; radical surgery was not recommended, considering his age, end-stage kidney disease, and performance status at that time. He was about to undergo hemodialysis, but the conduit necrosis developed before the start of hemodialysis.

Respiratory condition deteriorated within a week of hospitalization, and the patient died 2013 days after diagnosis. An autopsy was not performed in accordance with the family’s decisions.

## Discussion

The incidence of GCN has been reported to be 2% in a series of 1000 patients [2] who underwent esophagectomies at the University of Pittsburgh Medical Center. Peripheral arterial disease, impaired cardiac function, poor nutritional status, and diabetes mellitus are presumed risk factors for this devastating condition [3]. However, no reliable preoperative risk assessment has yet been established [4]. Recently, indocyanine green staining has been recommended for the intraoperative evaluation of the blood flow of the conduit [5], and delayed reconstruction is recommended if ischemic conditions are suspected in the conduit [6]. In our case, moderate to severe chronic kidney disease (glomerular filtration rate 39.1 mL/min) and old age were listed as risk factors. GCN was classified by Veeramootoo et al., and for massive necrosis graded as grade 3 in this classification, emergency surgical removal of the gangrenous portion of the conduit has been strongly recommended [7]. Delay in re-intervention may lead to devastating events such as fistulation of the trachea, lung, or thoracic wall [8–10]. Only a small number of patients have been reported to have undergone conservative treatment [11]. In our case, the anastomotic leakage showed no prolonged course, and no drainage was required within 2 weeks after the esophagectomy. In addition, endoscopic evaluation of the gastric conduit was unintentionally postponed because of additional adverse events, namely, rupture of the gallbladder and a hemorrhagic jejunal ulcer.

The GCN was not evident until POD 22 when the patient was stable and showed no sustained elevation of serum inflammatory markers. Follow-up endoscopy showed improved findings at every subsequent re-evaluation, and there were no cues to reconsider our strategy during the entire 9-month clinical course before the cure of conduit necrosis. However, the patient developed a lethal esophagopulmonary fistula more than 5 years after surgery. Delayed perforation of gastric conduit ulcer has been reported so far and associated with radiation therapy, NSAID use, or *Helicobacter pylori* infection [12–14]. The case presented here was free from such known risk factors. The endoscopic image of his anastomotic site ulcer is speculated as an ischemic one. His old age and deteriorating renal function would be listed as strong risk factors accounting for this rare condition.

Treatment of the GCN ultimately failed; however, his initial complication was managed after an in-hospital course of approximately 6 months.

Accurate and timely diagnosis of conduit necrosis is possible with routine endoscopic evaluation during the early postoperative course, which is recommended by several experts [15–17]. However, whether the early discovery of massive necrosis promises improved outcomes remains unclear because the success rate of radical surgery is unknown. Interestingly, Page et al. detected gastric conduit ischemia in 16 of 84 patients with an uneventful postoperative course, while 12 necrotic cases had no anastomotic leakage [17]. Three patients were considered to be doing well despite the endoscopic diagnosis of both necrosis and leakage and underwent surgery immediately after diagnosis. One patient died after re-operation. Several other retrospective studies compared the surgical or non-surgical strategy for anastomotic leakage and concluded controversial interpretations [18–20]. These studies included cases with less than severe gastric conduits, and one report [18] illustrated two cases of massive conduit necrosis necessitating esophageal diversion; one died before reconstruction surgery, and the other required kidney transplantation.

## Conclusions

Although our experience casts doubt on the classical belief that urgent surgery to remove the necrotic conduit is definitively safer than conservative treatment, we nonetheless believe that emergency radical surgery should be recommended for massive GCN if discovered early. Our clinical experience would be worth reporting, as the endoscopic images demonstrate the curative process of such massive conduit necrosis.

## Data Availability

Not applicable.

## Abbreviations

ALT: Alanine aminotransferase
AST: Aspartate aminotransferase
BUN: Blood–urea–nitrogen
CT: Computed tomography
CEA: Carcinoembryonic antigen
CA19-9: Carbohydrate antigen 19-9
ESCC: Esophageal squamous cell carcinoma
GCN: Gastric conduit necrosis
POD: Postoperative day
T-bil: Total bilirubin
